# Prognostic significance of peripheral monocyte count in patients with extranodal natural killer/T-cell lymphoma

**DOI:** 10.1186/1471-2407-13-222

**Published:** 2013-05-03

**Authors:** Jia-Jia Huang, Ya-Jun Li, Yi Xia, Yu Wang, Wen-Xiao Wei, Ying-Jie Zhu, Tong-Yu Lin, Hui-Qiang Huang, Wen-Qi Jiang, Zhi-Ming Li

**Affiliations:** 1 State Key Laboratory of Oncology in South China, Guangzhou, China; 2 Department of Medical Oncology, Sun Yat-sen University Cancer Center, 651 Dong Feng RD East, Guangzhou 510060, China

**Keywords:** Absolute monocyte count, Extranodal natural killer/T-cell lymphoma, Prognosis, Tumor microenvironment

## Abstract

**Background:**

Extranodal natural killer/T-cell lymphoma (ENKL) has heterogeneous clinical manifestations and prognosis. This study aims to evaluate the prognostic impact of absolute monocyte count (AMC) in ENKL, and provide some immunologically relevant information for better risk stratification in patients with ENKL.

**Methods:**

Retrospective data from 163 patients newly diagnosed with ENKL were analyzed. The absolute monocyte count (AMC) at diagnosis was analyzed as continuous and dichotomized variables. Independent prognostic factors of survival were determined by Cox regression analysis.

**Results:**

The AMC at diagnosis were related to overall survival (OS) and progression-free survival (PFS) in patients with ENKL. Multivariate analysis identified AMC as independent prognostic factors of survival, independent of International Prognostic Index (IPI) and Korean prognostic index (KPI). The prognostic index incorporating AMC and absolute lymphocyte count (ALC), another surrogate factor of immune status, could be used to stratify all 163 patients with ENKL into different prognostic groups. For patients who received chemotherapy followed by radiotherapy (102 cases), the three AMC/ALC index categories identified patients with significantly different survivals. When superimposed on IPI or KPI categories, the AMC/ALC index was better able to identify high-risk patients in the low-risk IPI or KPI category.

**Conclusion:**

The baseline peripheral monocyte count is shown to be an effective prognostic indicator of survival in ENKL patients. The prognostic index related to tumor microenvironment might be helpful to identify high-risk patients with ENKL.

## Background

Extranodal nasal-type natural killer/T-cell lymphoma (ENKL) is a rare lymphoid neoplasm characterized by a cytotoxic phenotype. It is associated with Epstein–Barr virus (EBV) infection, vascular destruction, and obvious necrosis [1,2]. Its incidence is low in western populations but more prevalent in Latin America and East Asia, including China [3-5]. ENKL in China accounts for approximately 5–16% of all lymphoid neoplasms [5-8]. Patients with ENKL have heterogeneous clinical manifestations and prognosis [9,10]. Using the International Prognostic Index (IPI), nearly 80% of ENKL cases were categorized as low-risk, although some patients in this category have a poor prognosis [11]. Therefore, more and more studies are trying to find novel prognostic markers or another index, such as the Korean prognostic index (KPI), to better stratify patients with ENKL based on risk [12].

Immune system deficiency is a risk factor for non-Hodgkin lymphomas (NHL) [13,14]. Gene-expression profiling studies show an interaction between the host immune system and lymphoma [15-17]. Lymphopenia, a surrogate factor of immune status, is reported to be an unfavorable prognostic factor for leukemia, B-cell neoplasms [18-22]. In the recent preclinical studies, monocytes are found to be plentiful in the tumor microenvironment and contribute to the growth of malignant T-cells [23]. Monocytes have also been found to induce the proliferation of natural killer (NK)/T lymphoma cells and to infiltrate lymphomas [24]. Recent evidence shows a relationship between peripheral monocyte count (another immunologically relevant marker) and survival in patients with diffuse large B-cell lymphoma (DLBCL) and follicular lymphoma (FL) [25,26]. To the best of our knowledge, the prognostic impact of absolute monocyte count (AMC) in NK/T cell lymphoma has never been assessed. The current study was performed to investigate the prognostic influence of monocytes (another surrogate marker for tumor microenvironment) on survival in ENKL, and provide some information on host immunity at diagnosis for better risk stratification in patients with ENKL.

## Methods

### Ethics statement

This study was approved by the Institutional Review Board (IRB) of Sun Yat-Sen University Cancer Center. Study was performed in accord with the principles of the Declaration of Helsinki. All patients agreed to use their medical records for research.

### Patients and staging

This retrospective study included 163 patients with ENKL newly diagnosed between January 2001 and December 2009 at the Sun Yat-Sen University Cancer Center of China. All the cases were pathologically confirmed as extranodal NK/T-cell lymphoma, nasal type, based on morphologic and immunophenotypic criteria of the 2001 World Health Organization (WHO) classification. Cases with blastic NK-cell lymphoma/leukemia, aggressive NK-cell lymphoma/leukemia, peripheral T-cell lymphoma, unspecified, or with negative EBV *in situ* hybridization, were excluded. Informed consent was obtained from all patients prior to treatment.

Information regarding demographics, Eastern Cooperative Oncology Group (ECOG) performance status, physical examinations, systemic B symptoms, complete blood count (CBC), biochemical profiles, and serum lactate dehydrogenase (LDH) level were collected for analysis. The staging was based on the Ann Arbor staging system. Whole-body positron emission tomography/computed tomography (PET/CT) scans, CT scans or magnetic resonance imaging (MRI) scans of the involved sites, as well as thorax, abdomen, and pelvic CT scans, and bone marrow findings were used for staging. Patients with contiguous involvement extending to the adjacent tissues or organs were regarded as stage IE. Upper aerodigestive tract NK/T-cell lymphoma (UNKTL) and extra- upper aerodigestive tract NK/T-cell lymphoma (EUNKTL) were defined as previously described [8,27]. The International Prognostic Index (IPI: stage, ECOG performance status, serum LDH, stage, extranodal sites) and the Korean Prognostic Index for NK/T-cell lymphoma with nasal disease (KPI: serum LDH level, B symptoms, stage, regional lymph nodes involvement) were evaluated as previously described [12,28].

### Treatment modalities and response criteria

Patients received one of the following treatments: (i) chemotherapy followed by radiotherapy; (ii) chemotherapy alone; (iii) involved-field radiation alone; (iv) surgery followed by radiation; (v) best supportive care. The first-line chemotherapy regimens were: EPOCH (etoposide, doxorubicin, vincristine, cyclophosphamide, prednisone), CHOP (cyclophosphamide, doxorubicin, vincristine, and prednisone) or CHOP-like therapy, alternating triple therapy (CHOP-B [cyclophosphamide, doxorubicin, vincristine, prednisone, bleomycin], IMVP-16 [ifosfamide, etoposide, methotrexate], and DHAP [dexamethasone, cytarabine, cisplatin]), GEMOX (gemcitabine, oxaliplatin) +/− L-asparaginase, and SMILE (dexamethasone, methotrexate, ifosfamide, L-asparaginase, etoposide). Radiotherapy for the involved-field was given in daily fractions of 2 Gy (five fractions per week) for a total of 36–68 Gy. The International Working Group Recommendations for Response Criteria for non-Hodgkin’s lymphoma were used to evaluate the treatment response [29].

### Statistical analysis

Overall survival (OS) and progression-free survival (PFS) were assessed by the Kaplan–Meier method. OS was calculated from the date of diagnosis to the date of death from any cause, or date of the last follow-up. PFS was calculated from the date of diagnosis to the date of first lymphoma progression, death from any cause, or date of the last follow-up [29]. The absolute monocyte count (AMC) at diagnosis was analyzed as both continuous and dichotomized parameters. The optimal cut-off values of the AMC were determined using the receiver operating characteristics (ROC) method. In the ROC curve analysis, survival outcomes were dichotomized into death *versus* survival. The relationship between AMC (as a dichotomized variable) and clinical parameters was analyzed by Pearson's *χ*2 test. Survival curves were constructed using the Kaplan–Meier method. The prognostic influence of different parameters on survival was established by multivariate analysis using the Cox proportional hazards model. The two-tailed log-rank test method was used to determined statistical difference, and *P* value of less than 0.05 was regarded as statistically significant. Statistical analysis was carried out by SPSS 16.0 software.

## Results

### Patients characteristics

The baseline characteristics of all 163 patients with ENKL are shown in Table 1. The median age at the time of diagnosis was 43 years (range: 17–80). There was a male predominance (113 men and 50 women). Eight-five patients (52.1%) presented systemic B symptoms, and 55 patients (33.7%) had elevated serum LDH level prior to treatment. Nearly 80% of the patients (128 cases) had localized disease (Ann Arbor stage: I-II). Upper aerodigestive tract NK/T-cell lymphoma (UNKTL) was diagnosed in 140 cases (85.9%) and extra-upper aerodigestive tract NK/T-cell lymphoma (EUNKTL) in 23 cases (14.1%). For those with EUNKTL, the most common sites of primary extranodal involvement were cutaneous or soft tissue (11 cases), followed by the gastrointestinal tract (5 cases). International Prognostic Index (IPI) assessment found that the majority of the patients (125 cases, 76.7%) were in the low-risk category (IPI score = 0–1). The Korean Prognostic Index (KPI) was assessable in 138 patients with nasal disease. In the KPI model, 84 patients (60.9%) had no or one adverse factor, and the remaining 54 patients (39.1%) had at least two adverse factors.

**Table 1:** Clinical characteristics of 163 patients with extranodal nasal-type natural killer/T-cell lymphoma (ENKL) at diagnosis

**Characteristics**	**Number of patients**	**%**
Age, median y (range)	43 (17–80)	
Sex		
Male	113	69.3
Female	50	30.7
ECOG score		
0-1	151	92.6
≥ 2	12	7.4
Serum LDH > 245 u/l	55	33.7
B symptoms	85	52.1
Extranodal sites of involvement ≥ 2	23	14.1
Ann Arbor Stage		
I-II	128	78.5
III-IV	35	21.5
Subtype		
UNKTL	140	85.9
EUNKTL	23	14.1
Regional lymph node involvement^a^	53	38.4
KPI score^b^		
0-1	84	60.9
2-4	54	39.1
IPI score		
0-1	125	76.7
2-5	38	23.3
AMC ≥ 0.50×10^9^/L	118	72.4

Abbreviations: ECOG PS, Eastern Cooperative Oncology Group performance status; LDH, lactate dehydrogenase; UNKTL, upper aerodigestive tract NK/T-cell lymphoma; EUNKTL, extraupper aerodigestive tract NK/T-cell lymphoma; KPI, Korean Prognostic Index; IPI, International Prognostic Index; AMC, absolute monocyte count.

^a^ Complete data on regional lymph node involvement was available in 138 patients.

^b^ Complete data on KPI prognostic score was assessable in 138 patients.

The AMC were obtained from the complete blood count (CBC) test prior to treatment. The median AMC at diagnosis, range, and the 25% and 75% quartiles were 0.60×10^9^/L, 0.05–2.10×10^9^/L, 0.44×10^9^/L, and 0.80×10^9^/L, respectively. The most discriminative cut-points of the AMC was 0.495×10^9^/L (area under the curve [AUC]: 0.630, 95% confidence interval: 0.534-0.726, *P* = 0.011), as determined by receiver operating characteristics (ROC) analysis. Therefore, AMC ≥0.50×10^9^/L which were close to the most discriminative cut-points, were selected as the optimal cut-off values. The most discriminative cut-points of the ALC were 1.145×10^9^/L (AUC: 0.601, 95% confidence interval: 0.512-0.690, *P* = 0.027), as determined by ROC analysis. Therefore, ALC ≤1.10×10^9^/L, which was close to the most discriminative cut-point, was selected as the optimal cut-off value. Patients with high AMC at diagnosis tended to have poorer performance status (P = 0.002) and IPI >1 (P = 0.022).

### Survival and prognostic factors

The initial treatment modalities were chemotherapy followed by radiotherapy (n = 102); chemotherapy alone (n = 50); surgery followed by radiation (n = 2); radiotherapy alone (n = 4); and supportive care (n = 5). The data on evaluation of treatment response to the initial therapy were available in 151 cases (92.6%). Complete remission (CR) was observed in 71 patients (47.0%). No statistical difference was found in the baseline clinical characteristics between patients with AMC ≥0.50×10^9^/L and those with AMC <0.50×10^9^/L.

**Figure 1:**
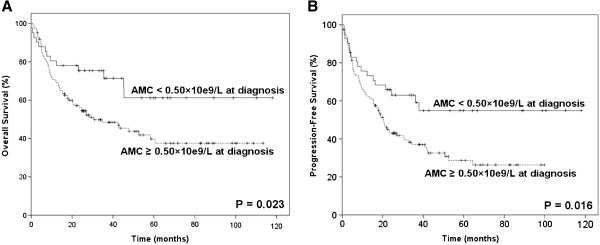
**Survivals in ENKL stratified by AMC.** Overall survival (**A**) and progression-free survival (**B**) of all patients (n=163) with ENKL stratified by the absolute monocyte count (≥0.50×10^9^/L *versus* < 0.50×10^9^/L) at diagnosis.

The estimated overall survival (OS) and progression-free survival (PFS) at 5 years was 43.7% and 33.9%, respectively. Patients with higher AMC level (AMC ≥0.50×10^9^/L, n = 118) seemed to have shorter OS and PFS (OS: *P* = 0.023; PFS: *P* = 0.016; Figure 1A and B). Univariate analysis of AMC as a continuous variable also showed AMC to be related to inferior survival (OS: *P* <0.001; PFS: *P* = 0.049). As KPI and IPI are commonly used prognostic indexes for ENKL, they were included with AMC in multivariate analysis. As shown in Table 2 and Table 3, AMC retained their prognostic impact on OS and PFS in patients with ENKL, independent of IPI score. KPI score was not found to be an independent prognostic factor of survival in multivariate analysis.

**Table 2:** Multivariate Cox-regression analysis of variables related to overall survival in patients with extranodal nasal-type natural killer/T-cell lymphoma (n=163)

**Variables**	**Hazard ratio (HR)**	**95% Confidence index (CI)**	***P***
AMC ≥ 0.50×10^9^/L	2.124	1.141-3.956	0.018
IPI score	2.197	1.415-3.409	<0.001

Abbreviations: AMC, absolute monocyte count; IPI, International Prognostic Index.

**Table 3:** Multivariate Cox-regression analysis of variables related to progression-free survival in patients with extranodal nasal-type natural killer/T-cell lymphoma (n=163)

**Variables**	**Hazard ratio (HR)**	**95% Confidence index (CI)**	***P***
AMC ≥ 0.50×10^9^/L	1.906	1.127-3.223	0.016
IPI score	1.728	1.163-2.568	0.007

Abbreviations: AMC, absolute monocyte count; IPI, International Prognostic Index.

### The AMC/ALC prognostic index identifies high-risk patients in ENKL

**Figure 2:**
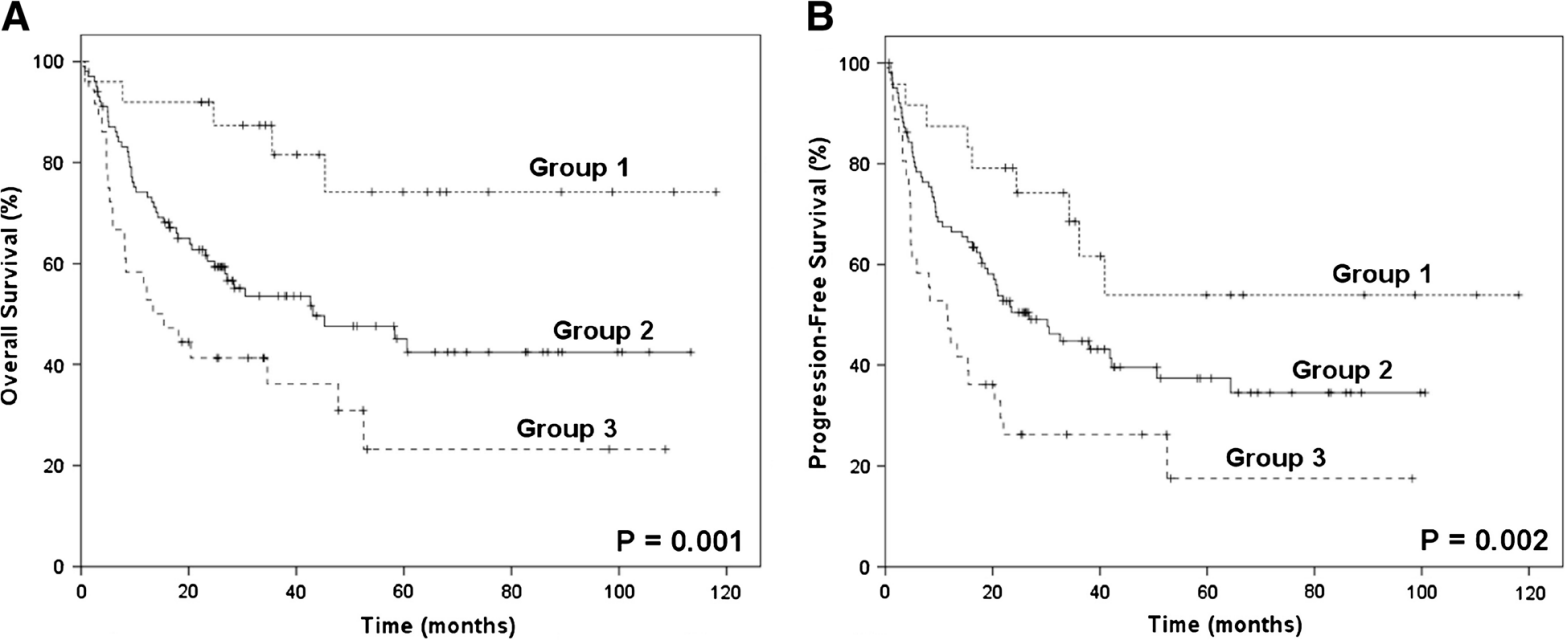
**Survivals in ENKL categorized by the prognostic index.** Overall survival (**A**) and progression-free survival (**B**) of all patients (n=163) with ENKL categorized by the absolute monocyte and lymphocyte prognostic index. Group 1 (low risk), AMC <0.50×10^9^/L and ALC>1.10×10^9^/L; Group 2 (intermediate risk), AMC ≥ 0.50×10^9^/L or ALC ≤ 1.10×10^9^/L; Group 3 (high risk), AMC ≥ 0.50×10^9^/L and ALC ≤ 1.10×10^9^/L.

**Figure 3:**
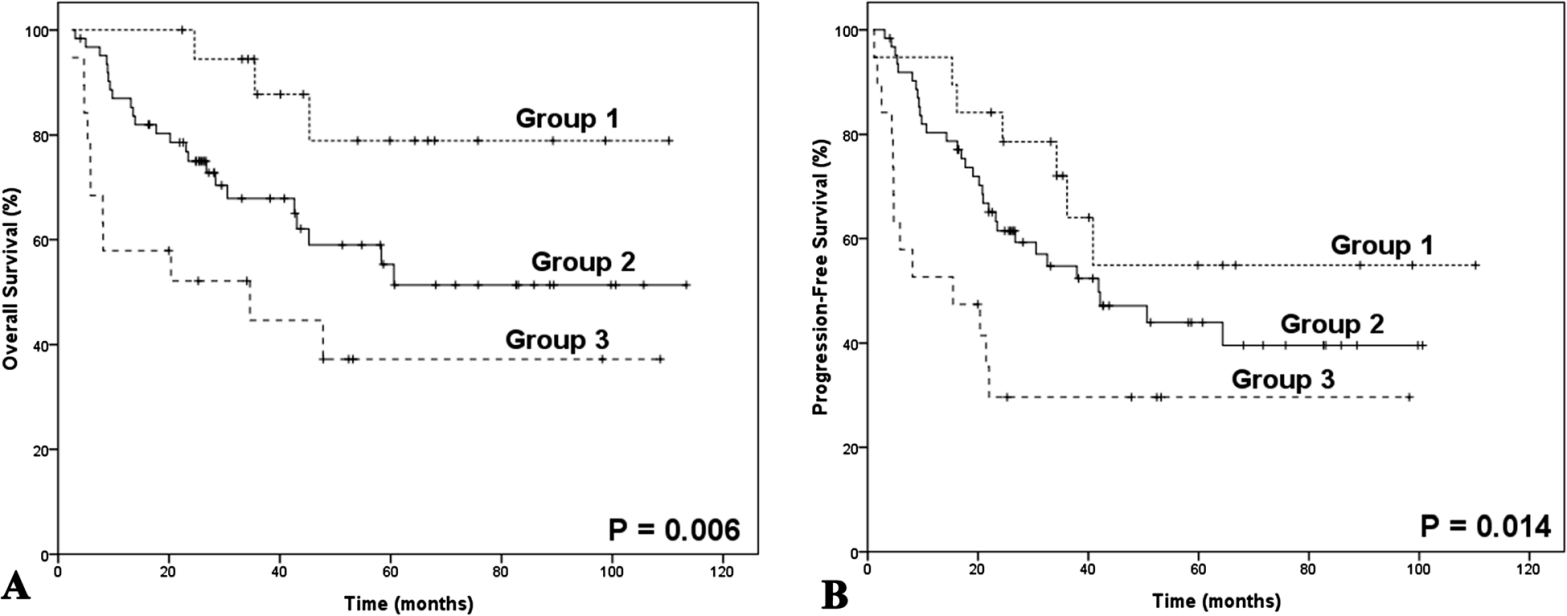
**Survivals in ENKL with chemotherapy followed by radiotherapy categorized by the prognostic index. **Overall survival (**A**) and Progression-free survival (**B**) of patients received chemotherapy followed by radiotherapy (n=102) categorized by the absolute monocyte and lymphocyte prognostic index. Group 1 (low risk), AMC <0.50×10^9^/L and ALC>1.10×10^9^/L; Group 2 (intermediate risk), AMC ≥ 0.50×10^9^/L or ALC ≤ 1.10×10^9^/L; Group 3 (high risk), AMC ≥ 0.50×10^9^/L and ALC ≤ 1.10×10^9^/L.

**Figure 4:**
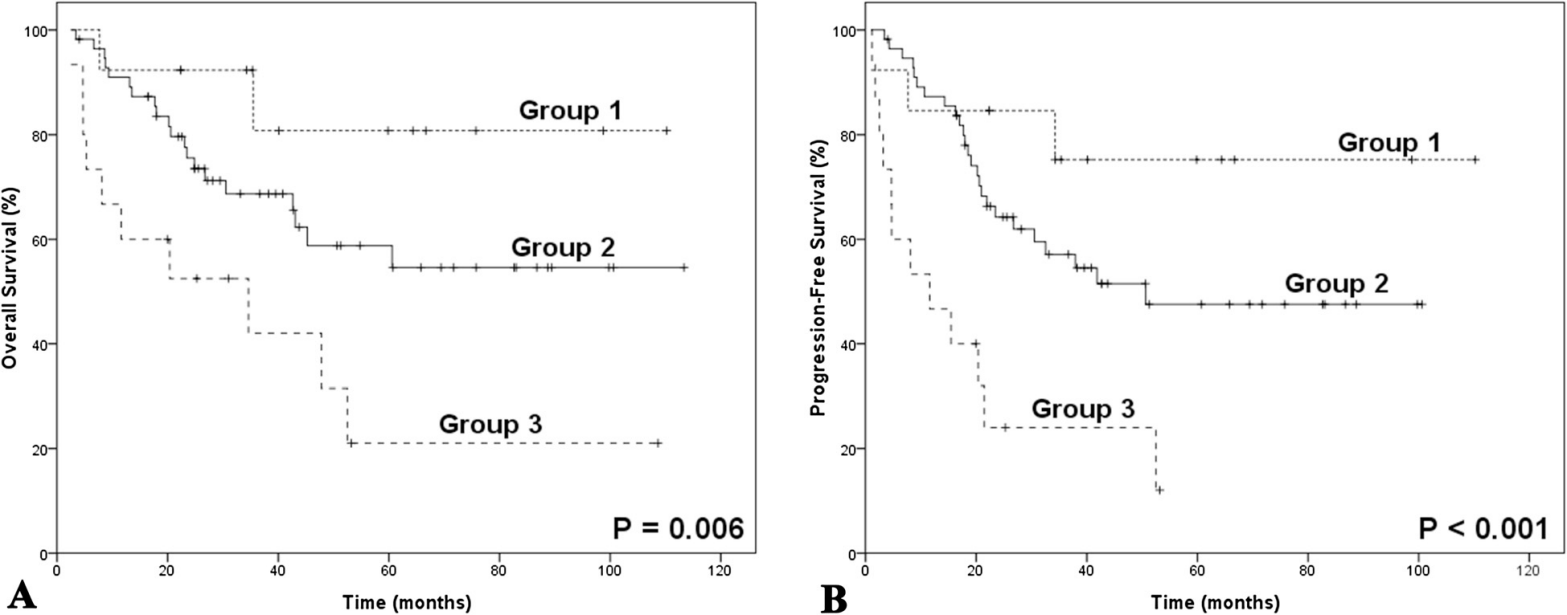
**Survivals in ENKL with low-risk KPI stratified by the prognostic index. **Overall survival (**A**) and progression-free survival (**B**) of patients with low-risk KPI score (KPI score = 0–1, n=84) as determined by the absolute monocyte and lymphocyte prognostic index. KPI, Korean Prognostic Index. Group 1 (low risk), AMC <0.50×10^9^/L and ALC>1.10×10^9^/L; Group 2 (intermediate risk), AMC ≥ 0.50×10^9^/L or ALC ≤ 1.10×10^9^/L; Group 3 (high risk), AMC ≥ 0.50×10^9^/L and ALC ≤ 1.10×10^9^/L.

**Figure 5:**
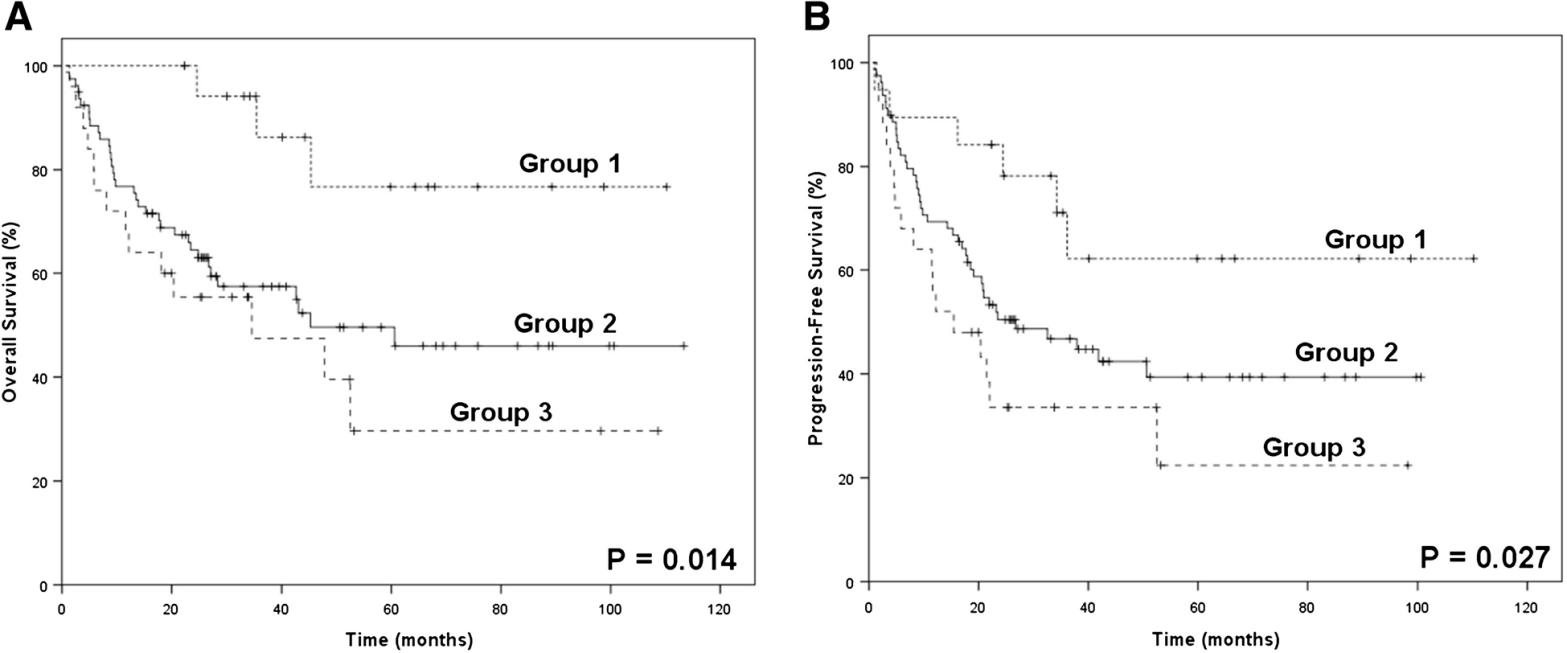
**Survivals in ENKL with low-risk IPI stratified by the prognostic index.** Overall survival (**A**) and progression-free survival (**B**) of patients with low-risk IPI score (IPI score = 0–1, n=125) as determined by the absolute monocyte and lymphocyte prognostic index. IPI, International Prognostic Index. Group 1 (low risk), AMC <0.50×10^9^/L and ALC>1.10×10^9^/L; Group 2 (intermediate risk), AMC ≥ 0.50×10^9^/L or ALC ≤ 1.10×10^9^/L; Group 3 (high risk), AMC ≥ 0.50×10^9^/L and ALC ≤ 1.10×10^9^/L.

Absolute lymphocyte count (ALC), as an indicator of immune status, was shown to be associated with OS and PFS in our previous study [19]. However, either AMC or ALC, as a single parameter, appeared to have limited ability to identify patients in the poor-risk category. Therefore, we combined the baseline AMC with the baseline ALC as dichotomized variables, to obtain a host immunity-related prognostic index of survival in patients with ENKL. Regarding the AMC/ALC prognostic index as a categorical variable, all 163 patients were stratified into the following three risk categories: group 1 (low risk), AMC <0.50×10^9^/L and ALC>1.10×10^9^/L; group 2 (intermediate risk), AMC ≥0.50×10^9^/L or ALC ≤1.10×10^9^/L; group 3 (high risk), AMC ≥0.50×10^9^/L and ALC ≤1.10×10^9^/L. The associations between patients’ clinical characteristics and AMC/ALC prognostic index were evaluated. Patients with high risk AMC/ALC index seemed to have a higher rate of B symptoms (P = 0.005). The OS and PFS based on the AMC/ALC prognostic index for patients with ENKL are shown in Figure 2. Univariate analysis showed that the AMC/ALC index allowed the discrimination of the three risk groups with a 5-year OS ranging from 74.2% to 23.2%, *P* = 0.001 and a 5-year PFS varying from 54.0% to 17.5%, *P* = 0.002. For patients who received chemotherapy followed by radiotherapy (102 cases), the three AMC/ALC index categories identified patients with significantly different survivals (OS: *P* = 0.006; PFS: *P* = 0.014; Figure 3). Moreover, when applied to the patients with low-risk IPI score (IPI = 0–1) or low-risk KPI score (KPI = 0–1), the AMC/ALC index identified significantly different prognostic categories in patients with low-risk KPI score (OS: *P* = 0.006; PFS: *P* <0.001; Figure 4) and patients with low-risk IPI score (OS: *P* = 0.014; PFS: *P* = 0.027; Figure 5). Further stratification by AMC/ALC index category identified 15 patients (17.9%) in the low-risk KPI score category and 25 patients (20%) in the low-risk IPI score category as high risk (5-year OS of 21.0% and 29.7%, respectively).

## Discussion

Epidemiologic studies show that immune deficiency increases the risk of NHL [30,31]. The incidence of NHL was much higher in immunosuppressed individuals than in non-immunosuppressed individuals [32,33]. Gene-expression signature studies in patients with B-cell NHL indicated that the myeloid-lineage cells in the tumor microenvironment influence the survival outcomes of patients with DLBCL and FL [15,16]. Monocytes were found to heavily infiltrate tumor samples from lymphoma patients, and this infiltration was found to be related to tumor invasion and immune response suppression in the animal model of lymphoma [15]. Furthermore, monocytes were recently found to promote the cell growth and survival in T-cell lymphomas and NK/T-cell lymphoma [23,24]. The predictive role of absolute monocyte count has been studied in B-cell NHL, as well as some solid tumors [25,26,34-36]. Limited data are available about the influence of monocytes on the survival of patients with ENKL. Hence, our study was carried out to evaluate the relevance of the peripheral blood monocyte count (a surrogate marker of the tumor microenvironment and host immunity) as a prognostic marker of survival in ENKL.

In this series, relatively elevated AMC at diagnosis (when treated as a continuous or dichotomized variable) was related to inferior prognosis. In multivariate analysis, AMC retained its prognostic impact on survival outcome in ENKL, and was independent of the conventional prognostic index. Evidence that AMC is an adverse prognostic factor in NHL was recently provided by Wilcox et al. in patients with DLBCL and FL [25,26]. AMC combined with IPI or Follicular Lymphoma International Prognostic Index (FLIPI) score helped to identify patients with poor prognosis. In patients with some solid neoplasms such as melanoma, renal cell carcinoma, and small cell lung cancer, elevated monocytes used as an immune parameter were also related to unfavorable survival [34-37]. Myeloid-derived cells (MDCs), including tumor-infiltrating monocytes and their progeny, were demonstrated to promote tumor angiogenesis and metastasis by suppressing the host immune response through regulation of macrophage cytokine production [38]. In T-cell NHL, many MDCs are found in the tumor microenvironment. When malignant T cells are co-cultured with monocytes, the proliferation and growth of tumor cells are increased. In contrast, when monocytes are depleted, malignant T cell death is increased [23]. Similar phenomena are observed in NK/T-cell lymphoma. Monocytes promote the cell proliferation and growth of EBV-positive NK/T-cell lymphoma cells, as well as the EBV-encoded latent membrance protein-1 (LMP-1) expression and interferon inducible protein-10 (IP-10) production. These functions of monocytes are mediated by cell contact dependent interaction via interleukin (IL)-15 [24]. Production by NK/T-cell lymphoma cells of IP-10 (a major chemoattractant of human monocytes) can increase monocyte infiltration of the tumor microenvironment [39-41]. Therefore, there is a positive feedback relationship between monocytes and lymphoma cells in the microenviroment of NK/T-cell lymphoma [24].

Lymphocyte count is another surrogate marker of immune homeostasis, and lymphopenia is regarded as an indicator of immunoincompetence [42]. Lymphopenia was an independent prognostic factor of adverse survival in our previous studies [19]. In the current study, our results showed that monocyte was also helpful to predict prognosis in ENKL. Therefore, we devised a simple prognostic index related to tumor microenvironment and host immune status that incorporates both AMC and ALC. This index was independent of conventional prognostic indices (such as KPI and IPI) and can be easily applied in clinical practice. The AMC/ALC index stratified patients with ENKL into three risk categories with significantly different survival outcomes. The current study and previous studies showed that the majority of patients with ENKL have no or one adverse IPI score or KPI score [12,19]. When superimposed on the IPI or KPI score, the AMC/ALC index provides additional prognostic information. This novel index was able to identify about 20% of patients in the low-risk KPI or low-risk IPI score category as high risk (with an inferior 5-year OS of less than 30%).

The current management of newly diagnosed ENKL patients was mainly based on the involvement sites (localized or disseminated disease). In this series, although most of the ENKL patients with localized disease underwent chemotherapy combined with radiation, some patients in this subgroup had unfavorable prognosis. The AML/ALC index was helpful to identify patients with poor survival outcomes. Maybe the combined treatment modality is insufficient to cure the high-risk patients. Several retrospective studies suggested that high-dose chemotherapy followed by autologous hematopoietic stem cell transplantation (HD-AHSCT) showed promising results for patients with disseminated ENKL [43-45]. It is interesting to investigate the efficacy of HD-AHSCT in the high-risk patients with localized disease in future studies. In this series, the underlying positive or negative biases during the therapy or selection of patients were inevitable, due to the limitation of its retrospective nature. Future prospective studies are warranted to validate our results.

## Conclusion

In conclusion, the baseline monocyte count is helpful to predict prognosis in ENKL patients. The AMC/ALC prognostic index, incorporating variables associated with the tumor microenvironment and host immunity, may offer a better risk stratification for patients with ENKL, when superimposed on the IPI or KPI. This finding warrants confirmation in independent cohorts. Further investigation is required to achieve a better understanding of the mechanism underlying the relationship between immunology and prognosis in NK/T-cell lymphoma.

## Competing interests

The authors have declared that no competing interests exist.

## Authors’ contributions

JJH designed the study, participated in the statistical analysis, and drafted the manuscript. YJL performed the statistical analysis and participated in the collection of the clinical data. YX, YW, WXW, YJZ, TYL, HQH and WQJ participated in the collection of the clinical data. ZML conceived of the study, and participated in its design and coordination and helped to draft the manuscript. All authors read and approved the final manuscript.

## Pre-publication history

The pre-publication history for this paper can be accessed here:

http://www.biomedcentral.com/1471-2407/13/222/prepub
